# Effect of hemoglobin and oxygen saturation on adverse outcomes in children with tetralogy of fallot: a retrospective observational study

**DOI:** 10.1186/s12871-023-02290-y

**Published:** 2023-10-17

**Authors:** Qiao Liu, Xie Wu, Yinan Li, Hongbai Wang, Ran An, Dou Dou, Dongyun Bie, Yuan Jia, Su Yuan, Fuxia Yan, Jie Ding

**Affiliations:** Department of Anesthesiology, Fuwai Hospital, National Center of Cardiovascular Diseases, Chinese Academy of Medical Sciences and Peking Union Medical College, 167 Beilishi Road, Xicheng District, Beijing, 100037 China

**Keywords:** Tetralogy of Fallot (TOF), Hemoglobin, Oxygen saturation, Children, Outcome

## Abstract

**Background:**

Tetralogy of Fallot (TOF) is a common cyanotic congenital heart malformation that carries a high risk of right-to-left shunting. Anemia is characterized by decreased hemoglobin (Hb) levels that can affect tissue oxygen delivery and impact postoperative recovery in patients. Chronic hypoxia caused by right-to-left shunting of TOF could lead to compensatory increases in Hb to maintain systemic oxygen balance. This study aims to investigate whether preoperative Hb and blood oxygen saturation (SpO2) can predict adverse outcomes in children undergoing corrective surgery for TOF.

**Methods:**

This retrospective study included patients under 18 years of age who underwent corrective surgery for TOF at Fuwai Hospital between January 2016 and December 2018. Adverse outcomes, including in-hospital death, extracorporeal membrane oxygenation implantation, ICU stay > 30 days, and severe complications, were considered as the primary outcome. Univariable and multivariable logistic analyses were performed to identify independent risk factors for adverse outcomes. Propensity score-matched (PSM) analysis was also conducted to minimize the confounding factors.

**Results:**

A total of 596 children were included in the study, of which 64 (10.7%) experienced adverse outcomes. Hb*SpO2 < aaHb was identified as an independent risk factor for adverse outcomes (OR = 2.241, 95% CI = 1.276–3.934, P = 0.005) after univariable and multivariable logistic analyses. PSM analysis further confirmed the association between Hb*SpO2 < aaHb and adverse outcomes. Patients with Hb*SpO2 < aaHb had a significantly higher incidence of postoperative adverse outcomes, longer time of mechanical ventilation, and hospital stay, as well as higher in-hospital costs.

**Conclusions:**

Hb*SpO2 < aaHb is significantly associated with adverse outcomes in children undergoing corrective surgery for TOF. Clinicians can use this parameter to early identify high-risk children and optimize their postoperative management.

**Supplementary Information:**

The online version contains supplementary material available at 10.1186/s12871-023-02290-y.

## Background

Tetralogy of Fallot (TOF) is a common cyanotic congenital heart defect (CHD) and characterized by ventricular septal defect, pulmonary artery stenosis, overriding aorta, and right ventricular hypertrophy [1]. Despite the significant innovations and advancements in surgical technique, organ protection, and medical management that have made patients can benefit from TOF repair surgeries in the past decades, postoperative complications still impose a great burden on morbidity and mortality, including heart failure, malignant arrhythmia, low cardiac output syndrome, respiratory failure, severe renal failure and so on. Early identification of patients at high-risk of adverse outcomes is crucial for clinicians to provide comprehensive medical management and optimize resource allocation. Anemia is a common condition among patients undergoing cardiac surgery, decreased hemoglobin (Hb) levels can compromise tissue oxygen delivery, leading to tissue hypoxia and organ dysfunction [2]. Patients with TOF are at risk of right-to-left shunting, which can cause tissue hypoxia and present with cyanosis. Chronic hypoxia can trigger compensatory increases in Hb levels to maintain systemic oxygen homeostasis [3]. Preoperative Hb and SpO2 are all cheap and simple-to-obtain parameters in clinical routine. Therefore, we hypothesize that preoperative Hb and oxygen saturation (SpO2) levels may serve as predictors for postoperative adverse outcomes in children undergoing corrective surgery for TOF.

## Methods

This is a retrospective observational study and the report meets the requirements of Strengthening the Reporting of Observational Studies in Epidemiology (STROBE) statement (seen in Additional file 1). The study received approval from the Ethics Committee of the Chinese Academy of Medical Sciences Fuwai Hospital (NO.2023–2045), with a waiver of informed consent. All procedures were conducted in line with Fuwai Hospital regulations and guidelines. The methods conformed to the standards of the Institutional Ethics Committee, as well as the Declaration of Helsinki of 1964 and its amendments. The study included patients (age < 18 y) who underwent corrective surgery for TOF at Fuwai Hospital between January 2016 and December 2018. Patients were excluded if they met any of the following criteria: (1) missing data for preoperative Hb and SpO2; (2) confounding factors related to adverse outcomes, such as previous palliative surgery, complex cardiac defects (e.g., complete endocardial cushion defect, double-outlet right ventricle, right ventricular outflow tract stenosis, pulmonary hypertension), or combined with the genetic syndrome; (3) abnormal ventilation, including preoperative mechanical ventilation or ventilation with laryngeal mask during surgery; (4) emergency surgery. Data associated with surgeries were collected from the electric medical record system. The basic demographic data included gestational age, surgical age, sex, weight, height, body surface area (BSA), blood oxygen saturation (SpO2), cardiac defects, American Society of Anesthesiologists Physical Status Classification (ASA class), and blood routine test results. BSA was calculated by the following formulation: BSA = 0.0061 × height [cm] + 0.0128 × weight [kg] − 0.1529. The operative data included cardiopulmonary bypass (CPB) time, aortic cross-clamp (ACC) time, minimum temperature (T_min_) during CPB, blood loss, and transfusion and infusion volumes. All patients were followed up until discharge, researchers also need to record postoperative outcomes.

Chronic hypoxia can lead to a reactive increase of hemoglobin (Hb) to maintain the tissue oxygen supply. Since the physical dissolved oxygen is rare, we used non-invasive oxygen saturation (SpO2) to calculate arterial oxygen content (CaO2). The formulation of CaO2 is: CaO2(ml/dl) = 1.39(ml/g) × Hb(g/dl) × SpO2(%) ÷ 100. Due to the risk of right to left shunt for TOF patients, they might experience chronic hypoxia and exhibit cyanosis. We hypothesize that if preoperative Hb can sufficiently compensate for hypoxia and achieve an adequate CaO2 level (1.39 * Preoperative Hb * preoperative SpO2 ≥ 1.39 * Normal Hb * normal SpO2), the risk of postoperative adverse outcome could be significantly reduced. The normal Hb (aaHb) value varies by age, with the lower limits for neonatal hemoglobin being 14.5 g/dl, 2 months being 9 g/dl, 6 months being 10.5 g/dl, 2 years being 11.5 g/dl, adolescent girls being 12 g/dl, and adolescent boys being 13 g/dl. The normal range for SpO2 is 95–100%, the maximum value of SpO2 (100%) was used to calculate the normal CaO2. Finally, we simplified the formulation and hypothesized that preoperative Hb * SpO2 < aaHb could be associated with postoperative adverse outcomes in children with TOF.

The adverse outcome was selected as the primary outcome and which is a composite outcome, it was defined as in-hospital death, extracorporeal membrane oxygenation implantation, intensive care unit (ICU) > 30 days, severe complications that were life-threatening [4], including extubation failure, thromboembolic events, significant cardiac disorders, severe cerebrovascular accident, and severe renal failure. Extubation failure was defined as an extubation failure within 120 h after surgery or reintubation within 24 h after extubation. The secondary outcomes included mechanical ventilation time, acute kidney injury (AKI), length of ICU stay, postoperative hospital stay and hospital stay, and hospital costs. The definition of AKI was based on postoperative creatinine levels exceeding 1.5-fold of the baseline level.

### Statistical analysis

Continuous variables were presented as medians with 25th and 75th percentiles and compared by Mann-Whitney U test. Categorical variables were presented as frequencies and percentages and compared by Chi-squared test. Univariable and multivariable logistic regression analyses were performed to identify independent risk factors for adverse outcomes. As multivariate logistic regression typically requires a minimum of 5–10 positive events per variable [5, 6], this study has a sufficient sample size for the multivariate analysis. A collinearity test was conducted before multivariate analysis, and variables with tolerance < 0.1 or VIF > 10 show that there is a collinearity relationship between variables. Variables with a P-value < 0.1 in the univariable analysis or those deemed clinically relevant were included in the multivariable regression model using forward selection. Additionally, propensity score-matched (PSM) analysis was conducted to minimize the influence of confounding factors. The PSM was performed with a match tolerance of 0.01 and a matching ratio of 1:1 by nearest neighbor matching. All statistical analyses were performed by SPSS software version 25.0 (IBM, Armonk, NY, USA), and P-value < 0.05 was considered statistically significant.

## Results

**Fig. 1:**
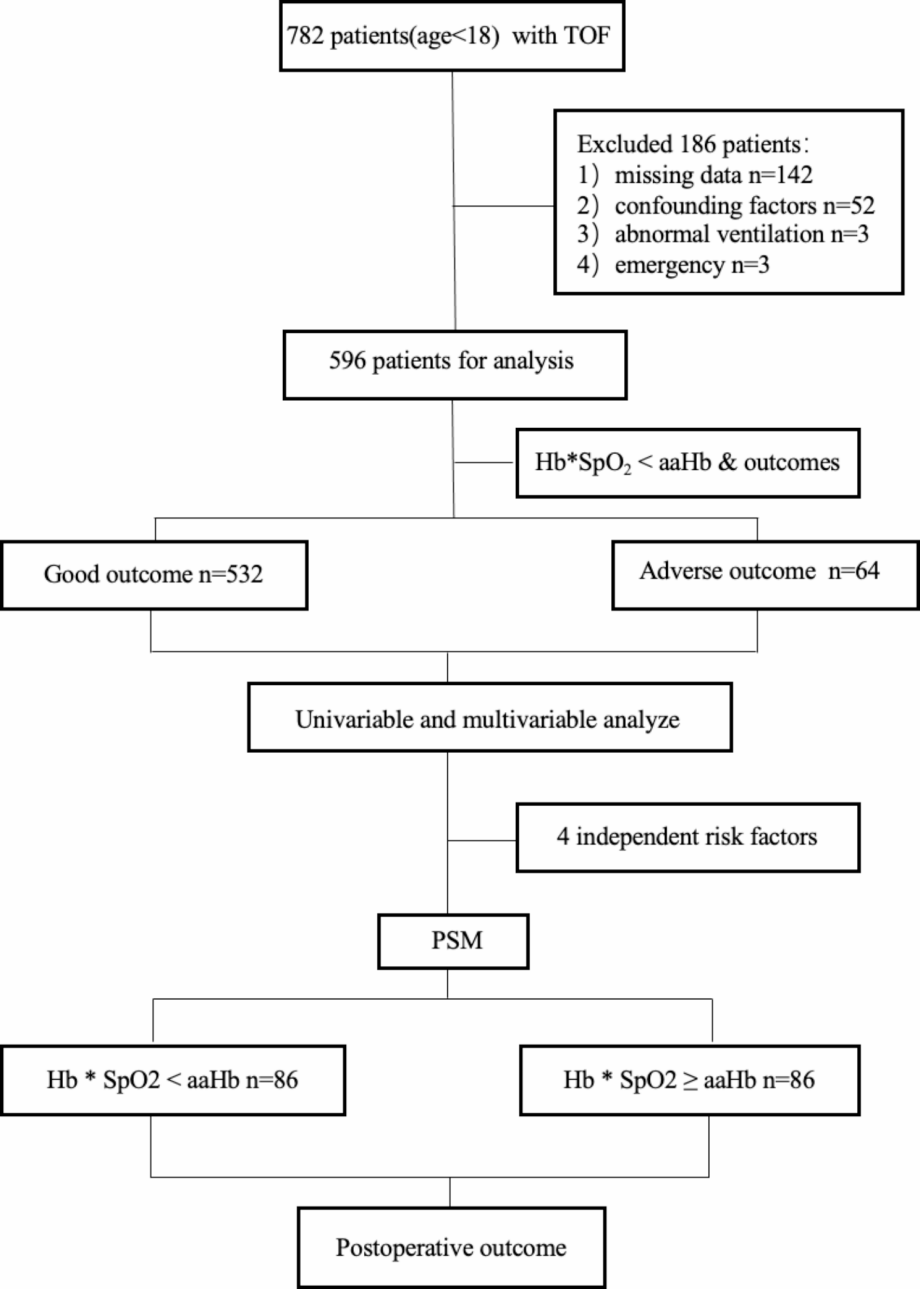
The Flowchart of the study TOF, Tetralogy of Fallot; Hb, hemoglobin; SpO2, blood oxygen saturation; aaHb, normal Hb; PSM, Propensity score-matched

The workflow of the study is depicted in Fig. 1. 782 children underwent corrective surgery for TOF in Fuwai Hospital during the past 3 years. After the exclusion criteria, 596 patients were included in this study, of which 64 patients were in adverse outcomes and 532 patients were in the normal group. Additional file 2 described a summary of adverse outcomes and 4(0.6%) died in the hospital. We divided patients into two groups according to Hb*SpO2 < aaHb and Hb < aaHb (Table 1), respectively, Hb*SpO2 < aaHb are associated with a higher incidence of adverse outcome (15.2% vs. 7.2%, P < 0.05).

**Table 1:** Association of preoperative hemoglobin with postoperative outcomes in patients

Adverse Outcomes	Hb < aaHb (n = 152)	Hb ≥ aaHb (n = 444)	P	Hb * SpO2 < aaHb (n = 263)	Hb * SpO2 ≥ aaHb (n = 333)	P
Death	1(0.7%)	1(0.2%)	> 0.999	3(1.1%)	1(0.3%)	0.326
ECMO placement	1(0.7%)	2(0.5%)	> 0.999	2(0.8%)	1(0.3%)	0.586
ICU > 30d	1(0.7%)	5(1.1%)	> 0.999	1(0.4%)	5(1.5%)	0.236
Extubation failure	8(5.3%)	18(4.15%)	0.498	16(6.1%)	10(3%)	0.072
Thromboembolic events	2(1.3%)	6(1.4%)	> 0.999	3(1.1%)	5(1.5%)	> 0.999
Significant cardiac disorders	5(3.3%)	7(1.6%)	0.194	8(3%)	4(1.2%)	0.112
Severe renal failure	3(2%)	14(3.2%)	0.58	11(4.2%)	6(1.8%)	0.09
Adverse outcomes	21(13.8%)	43(9.7%)	0.172	40(15.2%)	24(7.2%)	0.002^*^

Variables are expressed as frequency (percentage). ECMO, Extracorporeal Membrane Oxygenation; ICU, intensive care unit

To explore the relationship between Hb * SpO2 < aaHb and adverse outcomes after TOF corrective surgery, we divided patients into two groups based on adverse outcomes. Table 2 presented a descriptive result of all patients(n = 596), of which 363(60.9%) were male and 65(10.9%) were ASA class > III. The median surgical age was 9.6(6.9,14.7) months, the median weight was 8.5(7.5,10.0) kg, and the median height was 70(66,76) cm. The intraoperative results were as follows: 114 children underwent transannular patch(TAP) surgical technique and the others underwent valve-sparing technique, the median CPB and ACC time were 98(83,122) and 68(55,86) minutes, and with a minimum temperature of 30(28,30) °C. Compared with the patients with good outcomes, the patients with adverse outcomes were younger, shorter, weighed less, and had a lower BSA, SpO2, and absolute lymphocyte count, besides, they also had a longer CPB and ACC time, and lower minimum temperature. Importantly, patients with adverse outcomes had a higher incidence of Hb*SpO2 < aaHb than patients in the normal group (62.5% vs. 41.9%, P = 0.002).

**Table 2:** The demographic and perioperative information in patients

Variables	All patients (n = 596)	Good outcome (n = 532)	Adverse outcome (n = 64)	P
**Demographics**				
Gestational age (m)	39(38,40)	39(38,40)	38.6(38,40)	0.206
Age at surgery (m)	9.6(6.9,14.7)	9.7(6.9,15.2)	8.1(6.7,11.5)	0.04^*^
Sex (M/F)	363/233	320/212	43/21	0.276
Weight (kg)	8.5(7.5,10.0)	8.7(7.5,10)	8(7,9.2)	0.019^*^
Height (cm)	70(66,76)	70(66,76)	68.5(63.3,72.8)	0.026^*^
BSA (m^2^)	0.39(0.35,0.43)	0.39(0.35,0.43)	0.37(0.33,0.41)	0.018^*^
SpO_2_ < 90%	325(54.4%)	276(51.9%)	49(76.6%)	< 0.001^*^
ASA class > III	65(10.9%)	56(10.5%)	9(14.1%)	0.394
**Concomitant cardiac defects**				
ASD, n (%)	63(10.6%)	56 (10.5%)	7 (10.9%)	0.919
PDA, n (%)	62(10.2%)	50 (9.4%)	12 (18.8%)	0.021^*^
PFO, n (%)	168(28.2%)	155 (29.1%)	13 (20.3%)	0.138
PLSVC, n (%)	22(3.7%)	19 (3.6%)	3 (4.7%)	0.721
**Blood-routine parameter**				
WCC (10^9^/L)	9.59(7.99,11.88)	9.67(8.01,11.91)	9.23(7.61,11.59)	0.237
ANC (10^9^/L)	2.40(1.73,3.53)	2.38 (1.72, 3.49)	2.62 (1.77, 3.90)	0.213
ALC (10^9^/L)	6.03(4.55,7.76)	6.07(4.62,7.83)	5.4(3.75,7.21)	0.019^*^
AMC (10^9^/L)	0.57(0.44,0.74)	0.57 (0.44, 0.74)	0.57(0.45,0.74)	0.923
Hb (g/L)	13.0(11.3,14.6)	13.0(11.3,14.6)	12.9(10.8,14.7)	0.745
PLT (10^9^/L)	312(247,391)	314(253,390)	299(210,392)	0.113
Hb * SpO2 < aaHb	263(44.1%)	223(41.9%)	40(62.5%)	0.002^*^
**Intraoperative data**				
TAP/VS	114/482	90/442	24/40	< 0.001
CPB time (min)	98(83,122)	97(82,119)	114(94,141)	< 0.001^*^
ACC time (min)	68(55,86)	67(55,85)	80(63,102)	< 0.001^*^
T _min_ (℃)	30(28,30)	30 (28,30)	29 (28, 30)	0.005^*^
Blood loss (ml)	30(20,40)	30 (20, 40)	30 (20, 50)	0.578
Transfusion of RBC (ml)	30(20,40)	30 (20, 40)	30 (20, 50)	0.277
Infusion volume (ml)	60(50,80)	60 (50, 80)	65 (50, 85)	0.242

Continuous variables are presented as median with 25th and 75th; categorical variables are expressed as frequency (percentage). BSA, body surface area; SpO_2_, blood oxygen saturation; ASA class, American Society of Anesthesiologists Physical Status Classification; ASD, atrial septal defect; PDA, patent ductus arteriosus; PFO, patent foramen ovale; PLSVC, persistent left superior vena cava; WCC, white blood cell count; ANC, absolute neutrophil count; ALC, absolute lymphocyte count; AMC, absolute monocyte count; Hb, hemoglobin levels; PLT, platelet counts; TAP, transannular patch; VS, valve-sparing; CPB, cardiopulmonary bypass; ACC, aortic cross-clamp; T _min_, the minimum temperature; RBC, red blood cells

Variables with P < 0.1 in univariate analysis or the clinically relevant were included into the multivariate logistic analysis. After the collinearity diagnostics, we eventually included age, PDA, SpO_2_ < 90%, Hb*SpO2 < aaHb, CPB time, ACC time, T _min_, Hb, lymphocyte and TAP into the multivariable analysis. Finally, SpO2 < 90%, Hb*SpO2 < aaHb, ACC time, and TAP were significantly associated with adverse outcomes. The OR value and 95% confidence intervals (CI) of all variables were shown in Table 3.

**Table 3:** Univariable and multivariable logistic regression analyze

Variables	Univariable analysis		Multivariable analysis	
	OR (95%CI)	P	OR (95%CI)	P
Age (m)	0.981(0.957,1.007)	0.149		
PDA	2.225(1.114,4.444)	0.024		
SpO_2_ < 90%	0.943(0.924, 0.964)	<0.001	2.427(1.296,4.547)	0.006
Hb * SpO2 < aaHb	3.030(1.658,5.537)	< 0.001	2.241(1.276,3.934)	0.005
CPB time (min)	1.004(1.000,1.008)	0.067		
ACC time (min)	1.018 (1.008, 1.027)	< 0.001	1.017(1.007,1.027)	0.001
T _min_ (℃)	0.816 (0.710, 0.938)	0.004		
Hb (g/L)	1.001(0.991,1.010)	0.878		
ALC (10^9^/L)	0.875(0.779,0.982)	0.023		
TAP	2.947(1.692,5.130)	< 0.001	2.573(1.440,4.596)	0.001

OR, odds ratio; CI, confidence interval; PDA, patent ductus arteriosus; SpO_2_, blood oxygen saturation; Hb, hemoglobin; CPB, cardiopulmonary bypass; ACC, aortic cross-clamp; T _min_, the minimum temperature; ALC, absolute lymphocyte count; TAP, transannular patch

To further control the confounding factors, we conducted a one-to-one PSM, and 86 cases with Hb*SpO_2_ < aaHb were matched with 86 cases with Hb*SpO_2_ ≥ aaHb (Table 4). The standardized mean difference of covariates was less than 10% and showed a good PSM matching. The basic characteristics of patients were not statistically different after PSM. The adjusted cohort was used to compare the postoperative outcomes (Table 5). Patients with Hb*SpO2 < aaHb had a significantly higher incidence of adverse outcomes (15.1% vs. 4.7%, P < 0.05), meanwhile, the length of hospital stay and in-hospital cost were also statistically higher than those with Hb*SpO2 ≥ aaHb. Although the incidence of AKI between the two groups was not statistically significant, patients with Hb*SpO2 < aaHb were still nominally higher.

**Table 4:** The demographic and perioperative data before and after PSM

Variables	Before PSM			After PSM		
	Hb * SpO2 < aaHb (n = 263)	Hb * SpO2 ≥ aaHb (n = 333)	P	Hb * SpO2 < aaHb (n = 86)	Hb * SpO2 ≥ aaHb (n = 86)	P
Age (m)	9.1(6.9,12.9)	9.9(7,17.4)	0.038	9.6(7.2,17.4)	9.9(6.2,13.6)	0.337
PDA	32(12.2%)	30(9%)	0.21	8(9.3%)	10(11.6%)	0.618
SpO2 < 90%	174(66.2%)	151(45.3%)	< 0.001	43(50%)	51(59.3%)	0.22
Hb(g/L)	112(102,125)	140(130,157)	< 0.001	124(111,145)	135(122,141)	0.176
CPB time (min)	96(80,118)	100(85,124)	0.113	92(76,116)	106(80,132)	0.063
ACC time (min)	65(55,84)	70(56,88)	0.222	64(53,82)	70(54,94)	0.276
T min (℃)	30 (29, 30)	30 (28, 30)	0.057	30(29,31)	29(27,30)	0.126
TAP	46/217	68/265	0.402	12/74	15/71	0.529

Continuous variables are presented as median with 25th and 75th; categorical variables are expressed as frequency (percentage). PSM, propensity score matching; PDA, patent ductus arteriosus; SpO_2_, blood oxygen saturation; CPB, cardiopulmonary bypass; ACC, aortic cross-clamp; T _min_, the minimum temperature; TAP, transannular patch

**Table 5:** Outcomes of the patients in two groups after PSM

Outcome	Hb * SpO2 < aaHb (n = 86)	Hb * SpO2 ≥ aaHb (n = 86)	P
Adverse outcomes	13(15.1%)	4(4.7%)	0.021^*^
MV time (h)	19(10,25)	14.5(8,28)	0.348
AKI	17(19.8%)	12(14%)	0.309
LOIS (d)	2(1,4)	2(1,4)	0.756
LOHS (d)	15(12,19)	14(12,17)	0.046^*^
LOPS (d)	9(7,11)	9(7,12)	0.702
Cost (¥ 1000)	80.6(70.6,85.3)	72.8(63.7,84.4)	0.039^*^

Continuous variables are presented as median with 25th and 75th; categorical variables are expressed as frequency (percentage). MV, mechanical ventilation; AKI, acute kidney injury; LOIS, length of ICU stay; LOHS, length of hospital stay; LOPS, length of postoperative hospital stay

## Discussion

CHD is the most common congenital malformation and can be classified into cyanotic and non-cyanotic CHD. TOF is a typical example of cyanotic CHD, accounting for 7-10% [7]. With the progress of the disease, the obstruction of the right ventricular outflow tract worsened, the resistance of pulmonary circulation was greater than systemic circulation and which would induce the right to left shunt, allowing the unoxygenated blood flow directly enter the systemic circulation and resulting in cyanosis, SpO_2_ decreased, and systemic hypoxia [8]. Currently, corrective surgery is the primary treatment for TOF. Despite significant advancements in cardiac surgery technology, postoperative complications following TOF surgery remain a significant focus of perioperative management. Anemia is a common condition [9] among patients undergoing cardiac surgery and can impact oxygen-carrying capacity and tissue oxygenation [10], which would affect postoperative recovery. However, limited research has been conducted on anemia in pediatric CHD, and this study aims to explore whether preoperative anemia can predict adverse outcomes following pediatric corrective surgery for TOF.

As we know, Hb combines with oxygen of blood to form oxygenated Hb, which could transport oxygen to tissue cells and maintains a normal oxygen metabolic homeostasis. Due to the risk of right to left shunt in TOF patients, it would lead to cyanosis and chronic hypoxia as the condition worsen [11]. Chronic hypoxia triggers a compensatory increase of red blood cells and hemoglobin to maintain systemic oxygen balance [3]. Therefore, we hypothesize that if there is a reactive increase of preoperative Hb could compensate for hypoxia and maintain an oxygen balance, the hypoxic damage to systemic organs would be significantly reduced. Currently, there are no universally accepted criteria for anemia in children with CHD. Considering that the varying degrees of compensatory erythrocytosis and age can influence Hb levels in children, a fixed standard for anemia does not apply to CHD patients. The increase of red blood cell is negatively correlated with resting SpO2 [3, 12], Thus, we consider a preoperative Hb*preoperative SpO2 value lower than the normal Hb range as an indication of insufficient Hb concentration to maintain adequate oxygen supply and could be considered as anemia.

Several studies have demonstrated that preoperative anemia can increase morbidity and mortality, prolong ICU and hospital stay times in adult cardiac surgery [13, 14]. The pathophysiology basis of anemia is the reduction of oxygen-carrying capacity for Hb, which can result in tissue hypoxia and organ dysfunction [15]. Karkouti et al. found that anemia can affect the renal oxygen supply, aggravate oxidative stress reactions, and ultimately lead to acute kidney injury [16]. In our study, although the incidence of AKI was not statistically significant, patients with Hb*SpO2 < aaHb still exhibited a nominally higher incidence than those with Hb*SpO2 ≥ aaHb (19.8% vs. 14.0%, Table 5). Apart from as a new standard for anemia in TOF children, Hb*SpO2 < aaHb can also assist to reflect the balance of oxygen supply and demand. Under normal physiological conditions, oxygen delivery and consumption maintain a dynamic equilibrium [17]. When oxygen delivery gradually decreases to a certain extent, oxygen consumption also decreases, which can exacerbate hypoxic damage to systemic organs, particularly vulnerable tissues such as the brain, kidneys, and myocardial cells. Tissue oxygen delivery is directly related to CaO2, which primarily depends on Hb [18]. According to methodological descriptions, we used SpO2 to calculate CaO2(as mentioned above in Methods), and the simplified formulation of Hb*SpO2 < aaHb could reflect CaO2 is insufficient to maintain a systemic metabolic balance, which could increase the incidence of adverse outcomes, prolonged mechanical ventilation time, and hospital stay time. Moreover, anemia can trigger a series of compensatory reactions [19], such as tachycardia, vasodilation, increased cardiac output, etc. Patients who underwent cardiac surgery have a limited cardiac reserve capacity, the reduced oxygen delivery or increased oxygen consumption can lead to cardiac ischemia and elevate the risk of adverse events. Our results also confirmed this viewpoint in Table 5.

In this study, we retrospectively collected the perioperative data of children after corrective surgery for TOF. By comparing the relationship between preoperative Hb and clinical outcome, we found that Hb * SpO2 < aaHb was related to adverse outcomes (Table 1). Then we divided patients into two groups according to the postoperative outcome, Hb*SpO2 < aaHb was recognized as the independent risk factor for adverse outcomes after the univariable and multivariable logistic analysis (OR = 2.241, 95% CI = 1.276–3.934, P = 0.005). Finally, PSM was conducted to further reduce the influence of confounding factors, Hb * SpO2 < aaHb was significantly associated with a higher incidence of postoperative adverse outcomes (Table 5). This conclusion was consistent with other studies that preoperative anemia was linked with poor prognosis [14, 20]. Therefore, Hb * SpO2 < aaHb is suitable to early identify patients at high risk of adverse outcomes after TOF surgery. In addition, the elective repair surgery of TOF was proposed to conduct between 3 and 12 months of age [21], whereas there are 392(65.8%) children in the study under 12 months old, which may be due to the relatively poor economic level in our developing country and some patients only go to the hospital after experiencing severe symptoms. The in-hospital mortality for TOF surgery is 0.6% (Additional file 2) which is lower than other studies, which might be related to the strict inclusion and exclusion criteria and the fact that some patients died at home due to not receiving treatment for financial reasons.

Preoperative Hb*SpO2 < aaHb can early predict children at high-risk of adverse outcomes for corrective TOF surgery, optimizing SpO2 and Hb is important during perioperative management. For high-risk children, clinicians need to comprehensively evaluate their condition, engage in detailed preoperative discussions, and implement personalized medical management to optimize perioperative conditions. Furthermore, preoperative Hb*SpO2 < aaHb can also be an alterable factor during the perioperative period, clinicians can make efforts to optimize preoperative Hb levels before surgery, like limiting blood sampling and correcting coagulopathy to reduce blood loss, providing oral or intravenous iron to treat preoperative iron deficiency anemia, and even giving short-acting erythropoietin to further correct preoperative Hb levels if necessary. In addition, clinicians can also take measures to further optimize Hb, such as blood conservation techniques, sufficient hemostatic techniques, anticoagulant drugs to reduce bleeding, rational reduction of unnecessary intraoperative blood sampling to avoid blood waste and so on [23, 24]. Additionally, for children with severe hypoxia, clinicians can appropriately increase the partial pressure of oxygen to enhance tissue oxygen delivery. Nevertheless, prospective multicenter studies with larger sample sizes are necessary to determine whether the above measures can improve children’s prognosis after corrective surgery for TOF in the future.

### Limitations

The study also has some limitations. Firstly, this is a single-center study that could induce selection bias and the results may not be applicable to other centers. Secondly, due to the retrospective design, we are unable to collect all the detailed information and control all confounding factors, Thirdly, we only explored the short-term outcomes and did not make a long-term follow-up, therefore, the relationship between Hb*SpO2 < aaHb and long-term outcomes remains unclear.

## Conclusion

Preoperative Hb * SpO2 < aaHb is significantly associated with adverse outcomes for children undergoing corrective TOF surgery. Preoperative Hb * SpO2 is a cheap and simple-to-obtain variable and clinicians can utilize it to early identify children at high risk of poor prognosis.

### Electronic supplementary material

Below is the link to the electronic supplementary material.

Supplementary Material 1

Supplementary Material 2

## Data Availability

The datasets generated or analyzed during the current study are not publicly available due to data protection policy in our hospital but are available from the corresponding author on reasonable request.

## Abbreviations

aaHb: Normal Hb
ACC: Aortic cross-clamp
AKI: Acute kidney injury
ASA class: American Society of Anesthesiologists Physical Status Classification
BSA: Body surface area
CaO2: Arterial oxygen content
CHD: Congenital heart defect
CI: Confidence intervals
CPB: Cardiopulmonary bypass
Hb: Hemoglobin
ICU: Intensive care unit
PSM: Propensity score-matched
SpO2: Blood oxygen saturation
T_min_: Minimum temperature
TAP: Transannular patch
TOF: Tetralogy of Fallot

## References

[1] Wise-Faberowski L, Asija R, McElhinney DB (2019). Tetralogy of Fallot: everything you wanted to know but were afraid to ask. Paediatr Anaesth.

[2] Abeysiri S, Chau M, Richards T (2020). Perioperative Anemia Management. Semin Thromb Hemost.

[3] Broberg CS, Jayaweera AR, Diller GP, Prasad SK, Thein SL, Bax BE, Burman J, Gatzoulis MA (2011). Seeking optimal relation between oxygen saturation and hemoglobin concentration in adults with cyanosis from congenital heart disease. Am J Cardiol.

[4] Wu X, Luo Q, Su Z, Li Y, Wang H, Yuan S, Yan F (2021). Prognostic Value of Preoperative Absolute Lymphocyte Count in Children with tetralogy of Fallot. J Am Heart Assoc.

[5] Vittinghoff E, McCulloch CE (2007). Relaxing the rule of ten events per variable in logistic and Cox regression. Am J Epidemiol.

[6] Vergouwe Y, Steyerberg EW, Eijkemans MJ, Habbema JD (2005). Substantial effective sample sizes were required for external validation studies of predictive logistic regression models. J Clin Epidemiol.

[7] Forman J, Beech R, Slugantz L, Donnellan A (2019). A review of tetralogy of Fallot and Postoperative Management. Crit Care Nurs Clin North Am.

[8] Apitz C, Webb GD, Redington AN (2009). Tetralogy of Fallot Lancet.

[9] Fowler AJ, Ahmad T, Phull MK, Allard S, Gillies MA, Pearse RM (2015). Meta-analysis of the association between preoperative anaemia and mortality after surgery. Br J Surg.

[10] Dimopoulos K, Diller GP, Giannakoulas G, Petraco R, Chamaidi A, Karaoli E, Mullen M, Swan L, Piepoli MF, Poole-Wilson PA (2009). Anemia in adults with congenital heart disease relates to adverse outcome. J Am Coll Cardiol.

[11] Khatib I, Lebret E, Lambert V, Hascoet S (2017). Tetralogy of Fallot associated with multiple anomalies. Eur Heart J.

[12] Spence MS, Balaratnam MS, Gatzoulis MA (2007). Clinical update: cyanotic adult congenital heart disease. Lancet.

[13] Spadaccio C, Nenna A, Candura D, Rose D, Moscarelli M, Al-Attar N, Sutherland F (2022). Total arterial coronary artery bypass grafting in patients with preoperative anemia. J Card Surg.

[14] Dhir A, Tempe DK (2018). Anemia and patient blood management in Cardiac surgery-literature review and current evidence. J Cardiothorac Vasc Anesth.

[15] Jabagi H, Boodhwani M, Tran DT, Sun L, Wells G, Rubens FD (2019). The Effect of Preoperative Anemia on Patients undergoing cardiac surgery: a propensity-matched analysis. Semin Thorac Cardiovasc Surg.

[16] Karkouti K, Wijeysundera DN, Yau TM, Callum JL, Cheng DC, Crowther M, Dupuis JY, Fremes SE, Kent B, Laflamme C (2009). Acute kidney injury after cardiac surgery: focus on modifiable risk factors. Circulation.

[17] Mallat J, Rahman N, Hamed F, Hernandez G, Fischer MO (2022). Pathophysiology, mechanisms, and managements of tissue hypoxia. Anaesth Crit Care Pain Med.

[18] Nangaku M (2004). Hypoxia and tubulointerstitial injury: a final common pathway to end-stage renal failure. Nephron Exp Nephrol.

[19] Chaparro CM, Suchdev PS (2019). Anemia epidemiology, pathophysiology, and etiology in low- and middle-income countries. Ann N Y Acad Sci.

[20] Padmanabhan H, Siau K, Curtis J, Ng A, Menon S, Luckraz H, Brookes MJ (2019). Preoperative Anemia and outcomes in Cardiovascular surgery: systematic review and Meta-analysis. Ann Thorac Surg.

[21] Mimic B, Brown KL, Oswal N, Simmonds J, Hsia TY, Tsang VT, De Leval MR, Kostolny M (2014). Neither age at repair nor previous palliation affects outcome in tetralogy of Fallot repair. Eur J Cardiothorac Surg.

[22] Faraoni D, Meier J, New HV, Van der Linden PJ, Hunt BJ (2019). Patient blood management for neonates and children undergoing cardiac surgery: 2019 NATA Guidelines. J Cardiothorac Vasc Anesth.

[23] Gómez-Ramírez S, Bisbe E, Shander A, Spahn DR, Muñoz M (2019). Management of Perioperative Iron Deficiency Anemia. Acta Haematol.

[24] Warner MA, Shore-Lesserson L, Shander A, Patel SY, Perelman SI, Guinn NR (2020). Perioperative Anemia: Prevention, diagnosis, and Management throughout the Spectrum of Perioperative Care. Anesth Analg.

