# Supplementary material for: Effect of hemoglobin and oxygen saturation on adverse outcomes in children with tetralogy of fallot: a retrospective observational study
Source: BMC Anesthesiol. 2023 Oct 17;23:346. doi: 10.1186/s12871-023-02290-y (PMC10580598; doi:10.1186/s12871-023-02290-y)
Supplement: Supplementary file 1 — Supplementary Material 1 [file 12871_2023_2290_MOESM1_ESM.docx]

**Additional file 2 Summary of adverse outcomes**

| **Adverse Outcomes** | **Number (%)** |
| --- | --- |
| Death | 4(0.6%) |
| ECMO placement | 3 (0.5%) |
| ICU ＞ 30d | 6 (1%) |
| Severe complications |  |
| Extubation failure  Thromboembolic events  Significant cardiac disorders  Severe cerebrovascular accident  Severe renal failure | 26 (4.3%)  8 (1.3%)  12 (2%)  1 (0.1%)  17 (2.8%) |
| Total | 64 (10.9%) |

Variables are presented as n (%). ECMO, Extracorporeal Membrane Oxygenation; ICU, intensive care unit.
