# Supplementary material for: Effect of hemoglobin and oxygen saturation on adverse outcomes in children with tetralogy of fallot: a retrospective observational study
Source: BMC Anesthesiol. 2023 Oct 17;23:346. doi: 10.1186/s12871-023-02290-y (PMC10580598; doi:10.1186/s12871-023-02290-y)
Supplement: Supplementary file 2 — Supplementary Material 2 [file 12871_2023_2290_MOESM2_ESM.docx]

**Additional file 1.**

**STROBE Statement (checklist of items that should be included in reports of observational studies)**

|  | **Item**  **No.** | **Recommendation** |
| --- | --- | --- |
| **Title and abstract** | 1 | (a)Study design: a retrospective observational study.  (b)Informative and balanced summary: Chronic hypoxia caused by right-to-left shunting in TOF children could lead to compensatory increases in Hb to maintain systemic oxygen balance. This study aims to investigate whether preoperative Hb and SpO2 levels can predict adverse outcomes in children undergoing corrective surgery for TOF. This retrospective study included patients under 18 years of age who underwent corrective surgery for TOF. After statistical analyses, children with Hb*SpO2 < aaHb had a significantly higher incidence of postoperative adverse outcomes, longer time of mechanical ventilation, ICU stay, and hospital stay, as well as higher in-hospital costs. |
| **Introduction** |  |  |
| **Background/rationale** | 2 | For pediatric cardiac surgery, early identification of patients with poor prognoses is crucial for clinicians to provide comprehensive medical management and optimize resource allocation. Children with TOF are at risk of right-to-left shunting, which can cause tissue hypoxia and present with cyanosis. Chronic hypoxia triggers compensatory increases in Hb levels to maintain systemic oxygen homeostasis. We hypothesize that preoperative Hb and SpO2 levels may serve as predictors of postoperative adverse outcomes in children undergoing corrective surgery for TOF. |
| **Objectives** | 3 | The purpose of the study was to investigate whether preoperative Hb and SpO2 levels can predict adverse outcomes in children undergoing corrective surgery for TOF. |
| **Methods** |  |  |
| **Study design** | 4 | A retrospective observational study. |
| **Setting** | 5 | This study retrospectively collected patients(age<18y) who underwent corrective surgery for TOF at Fuwai Hospital between January 2016 and December 2018. |
| **Participants** | 6 | **Inclusion criteria:**1) under 18 years old; 2) underwent corrective surgery for TOF at Fuwai Hospital from January 2016 to December 2018  **Exclusion criteria**: 1) missing data for preoperative Hb and SpO2; 2) confounding factors related to adverse outcomes, such as previous palliative surgery, complex cardiac defects or combined with the genetic syndrome; 3) abnormal ventilation, including preoperative mechanical ventilation or ventilation with laryngeal mask during surgery; 4) emergency surgery. |
| **Variables** | 7 | **Primary Outcome:** Adverse outcome (in-hospital death, extracorporeal membrane oxygenation implantation, ICU >30 days, severe complications that were life-threatening, including extubation failure, thromboembolic events, significant cardiac disorders, severe cerebrovascular accident, and severe renal failure.)  **Secondary Outcomes:** ICU stay time, hospital stay time and postoperative hospital stay time, acute kidney injury, acute hepatic injury and hospitalization cost. |
| **Data sources**  **/ measurement** | 8 | **Data sources:** Electric medical record system.  **Measurement:**  BSA = 0.0061 × height [cm] + 0.0128 × weight [kg] - 0.1529  AKI was defined as postoperative creatinine levels exceeding 1.5-fold of the baseline level. |
| **Bias** | 9 | We collected the demographics, laboratory tests, and surgical associated information that could affected postoperative outcomes, then, we put the clinically relevant variables and variables with P<0.1 in univariable regression into multivariable regression model by forward variable selection to control the potential confounding factors. Finally, PSM was conducted to further minimize the influence of confounding factors |
| **Study size** | 10 | The multivariate logistic regression typically requires a minimum of 5-10 positive events per variable, this study has a sufficient sample size for the multivariate analysis. |
| **Quantitative variables** | 11 | Patients were divided into two groups based on postoperative adverse outcome. |
| **Statistical methods** | 12 | (a) Continuous variables were presented as medians with 25th and 75th percentiles and compared by Mann-Whitney U test. Categorical variables were presented as frequencies and percentages and compared by Chi-squared test. Univariable and multivariable logistic regression analyses were performed to identify independent risk factors for adverse outcomes. As multivariate logistic regression typically requires a minimum of 5-10 positive events per variable, this study has a sufficient sample size for the multivariate analysis. A collinearity test was conducted before multivariate analysis, and variables with tolerance < 0.1 or VIF > 10 show that there is a collinearity relationship between variables. Variables with a P-value < 0.1 in the univariable analysis or those deemed clinically relevant were included in the multivariable regression model using forward selection. Additionally, propensity score-matched (PSM) analysis was conducted to minimize the influence of confounding factors. The PSM was performed with a match tolerance of 0.01 and a matching ratio of 1:1 by nearest neighbor matching. All statistical analyses were performed by SPSS software version 25.0 (IBM, Armonk, NY, USA), and a P-value < 0.05 was considered statistically significant.  (b)Missing values were replaced by the mean values. |
| **Results** |  |  |
| **Participants** | 13 | (a) 782 children underwent corrective surgery for TOF in Fuwai Hospital during the past 3 years.  (b)We excluded patients with: a) missing data for Hb and SpO2(n=42); b) confounding factors related to adverse outcomes(n=52); c) abnormal ventilation(n=3); 4) emergency surgery(n=3).  (c)Finally, 596 patients were included in this study, of which 64 patients were in adverse outcomes and 532 patients were in the normal group.  (d)A recruitment figure has been added (**Figure 1**). |
| **Descriptive data** | 14 | (a) 60.9% children were male and 10.9% children were ASA class >III. The median surgical age was 9.6(6.9,14.7) months, the median weight was 8.5(7.5,10.0) kg, and the median height was 70(66,76) cm. The intraoperative results were as follows: 114 children underwent transannular patch (TAP) surgical technique and the others underwent valve-sparing technique, the median CPB and ACC time were 98(83,122) and 68(55,86) minutes, and with a minimum temperature of 30(28,30) °C.  (b)**Table 2** presents a summary of demographic data and perioperative variables of all patients. |
| **Outcome data** | 15 | 64 patients were in adverse outcomes and 532 patients were in the normal group.  Compared with the patients with good outcomes, the patients with adverse outcomes were younger, shorter, weighed less, and had a lower BSA, SpO2, and absolute lymphocyte count, besides, they also had a longer CPB and ACC time, and lower minimum temperature. Importantly, patients with adverse outcomes had a higher incidence of Hb*SpO2 < aaHb than patients in the normal group (62.5% vs 41.9%). |
| **Main results** | 16 | According to the univariable and multivariable regression results, we found that SpO2 < 90%, Hb*SpO2 < aaHb, ACC time, and TAP were independent risk factors for adverse outcome. The OR value and 95% confidence intervals (CI) of all variables were shown in **Table 3** |
| **Other analyses** | 17 | PSM was performed to further control the confounding factors, 86 cases with Hb*SpO2 < aaHb were matched with 86 cases with Hb*SpO2 ≥ aaHb. Patients with Hb*SpO2 < aaHb had a significantly higher incidence of adverse outcomes (15.1% vs 4.7%, P<0.05) and other outcomes. |
| **Discussion** |  |  |
| **Key results** | 18 | Preoperative Hb * SpO2<aaHb is significantly associated with adverse outcomes for children undergoing corrective TOF surgery. Preoperative Hb * SpO2 is a cheap and simple-to-obtain variable and clinicians can utilize it to early identify children at high risk of poor prognosis. |
| **Limitations** | 19 | Firstly, this is a single-center study that could induce selection bias and the results may not applicable to other centers. Secondly, due to the retrospective design, we are unable to collect all the detailed information and control all confounding factors, Thirdly, we only explored the short-term outcomes and did not make a long-term follow-up, therefore, the relationship between Hb*SpO2<aaHb and long-term outcomes remains unclear. |
| **Interpretation** | 20 | Anemia impact oxygen-carrying capacity and tissue oxygenation, which would affect postoperative recovery. However, limited research has been conducted on anemia in pediatric CHD. This study explored the relationship between Hb*SpO2<aaHb and postoperative outcomes. Preoperative Hb*SpO2 < aaHb can early predict adverse outcomes in children undergoing corrective TOF surgery. For high-risk children, clinicians need to comprehensively evaluate their condition, engage in detailed preoperative discussions, and implement personalized medical management to optimize clinical outcomes. Furthermore, preoperative Hb*SpO2 < aaHb can also be an alterable factor during the perioperative period. Clinicians can take measures to improve Hb levels during perioperative management. |
| **Generalisability** | 21 | The results came from a single-center retrospective study, prospective multicenter studies with larger sample sizes are necessary to further explore the relationship between Hb*SpO2 < aaHb and postoperative outcomes for children with TOF in the future. |
| **Other information** |  |  |
| **Funding** | 22 | There were no funding supporting in this study. |
